# Potent small molecule inhibitors against the 3C protease of foot-and-mouth disease virus

**DOI:** 10.1128/spectrum.03372-23

**Published:** 2024-03-11

**Authors:** Yunjeong Kim, Emma Pool, Eunji Kim, Chamandi S. Dampalla, Harry Nhat Nguyen, David K. Johnson, Scott Lovell, William C. Groutas, Kyeong-Ok Chang

**Affiliations:** 1 Department of Diagnostic Medicine and Pathobiology, College of Veterinary Medicine, Kansas State University, Manhattan, Kansas, USA; 2 Department of Chemistry, Wichita State University, Wichita, Kansas, USA; 3 Computational Chemical Biology Core, The University of Kansas, Lawrence, Kansas, USA; 4 Protein Structure and X-ray Crystallography Laboratory, The University of Kansas, Lawrence, Kansas, USA; Cornell University College of Veterinary Medicine, Ithaca, USA

**Keywords:** food-and-mouth disease, 3C protease, protease inhibitor, antiviral, structure-activity relationships

## Abstract

Foot-and-mouth disease (FMD) is one of the most devastating diseases of livestock which can cause significant economic losses, especially when introduced to FMD-free countries. FMD virus (FMDV) belongs to the family *Picornaviridae* and is antigenically heterogeneous with seven established serotypes. The prevailing preventive and control strategies are limited to restriction of animal movement and elimination of infected or exposed animals, which can be potentially combined with vaccination. However, FMD vaccination has limitations including delayed protection and lack of cross-protection against different serotypes. Recently, antiviral drug use for FMD outbreaks has increasingly been recognized as a potential tool to augment the existing early response strategies, but limited research has been reported on potential antiviral compounds for FMDV. FMDV 3C protease (3Cpro) cleaves the viral-encoded polyprotein into mature and functional proteins during viral replication. The essential role of viral 3Cpro in viral replication and the high conservation of 3Cpro among different FMDV serotypes make it an excellent target for antiviral drug development. We have previously reported multiple series of inhibitors against picornavirus 3Cpro or 3C-like proteases (3CLpros) encoded by coronaviruses or caliciviruses. In this study, we conducted structure-activity relationship studies for our in-house focused compound library containing 3Cpro or 3CLpro inhibitors against FMDV 3Cpro using enzyme and cell-based assays. Herein, we report the discovery of aldehyde and α-ketoamide inhibitors of FMDV 3Cpro with high potency. These data inform future preclinical studies that are related to the advancement of these compounds further along the drug development pathway.

**IMPORTANCE:**

Food-and-mouth disease (FMD) virus (FMDV) causes devastating disease in cloven-hoofed animals with a significant economic impact. Emergency response to FMD outbreaks to limit FMD spread is critical, and the use of antivirals may overcome the limitations of existing control measures by providing immediate protection for susceptible animals. FMDV encodes 3C protease (3Cpro), which is essential for virus replication and an attractive target for antiviral drug discovery. Here, we report a structure-activity relationship study on multiple series of protease inhibitors and identified potent inhibitors of FMDV 3Cpro. Our results suggest that these compounds have the potential for further development as FMD antivirals.

## INTRODUCTION

Foot-and-mouth disease (FMD) is a highly contagious disease affecting cloven-hoofed animals and one of the most devastating diseases with a significant economic impact. The classic signs of FMD in cattle and pigs include fever and vesicular lesions on the feet, mouth, and mammary glands, which result in severe production losses (reviewed in reference 1). Foot-and-mouth disease virus (FMDV) is a single-stranded, positive-sense RNA virus and belongs to the *Aphthovirus* genus within the family *Picornaviridae*. FMDV is antigenically heterogeneous, and seven serotypes are recognized worldwide: O, A, C, Asia1, Southern African Territories 1 (SAT), SAT2, and SAT3 (2). Furthermore, there is considerable antigenic diversity within each serotype with over 60 subtypes (1, 2). FMD is endemic in several parts of Asia and throughout most of Africa and the Middle East (2), but outbreaks sporadically occur in non-endemic areas and countries as FMDV is easily spread by aerosols and contact with contaminated materials and infected animals and animal products (1, 2). The United States has been FMD free without vaccination since 1929. However, there is a continuing risk of FMD transmission into the U.S. soil through international trades of animal and animal products (3). FMD outbreaks in the United States would have a catastrophic impact on the economy extending far beyond animal agriculture, and it is estimated that the impacts of FMD outbreaks in the United States would cost several to hundreds of billion dollars (4–6).

Effective control of FMD outbreaks requires rapid response due to a wide range of susceptible animals and the highly contagious nature of FMDV. Therefore, early detection, quarantine, and movement control are critical activities to limit FMD spread. Traditional FMD response strategies involve stamping out, which is depopulation of infected and in-contact susceptible animals, as an integral component, which can be combined with vaccination with or without subsequent depopulation of vaccinated animals depending on epidemiologic considerations (4). In countries that are free from FMD without vaccination, such as the US, stamping-out strategy alone may not sufficiently control FMD outbreaks, especially in densely populated areas, which may require FMD emergency vaccination (6). However, there are many challenges related to FMD emergency vaccination including delayed onset of protection, little or no cross-protection across serotypes and subtypes, and limitation of export from the loss of FMD-free without vaccination status. Therefore, antiviral agents are increasingly recognized as a potential measure, as supplemental or alternative to vaccination, to enhance emergency response system (7, 8).

Some direct-acting antivirals targeting viral polymerase RNA and viral RNA (RNA interference) and interferon or interferon-inducing agents against FMDV have been reported (9–24). However, only limited research is available on protease inhibitors that target FMDV (25–27). FMDV 3C protease (3Cpro) cleaves the viral polyprotein into mature, functional proteins during viral replication. The essential role of virus protease in replication and the high conservation of 3Cpro among different FMDV serotypes make it an excellent target for antiviral drug development. We have previously reported multiple series of protease inhibitors for important human and animal viruses that encode 3Cpro or 3C-like protease (3CLpro) (28–46). In this study, we conducted structure-activity relationship studies using our in-house focused compound library with the fluorescence resonance energy transfer (FRET) assay (35) and cell-based reporter assay for FMDV 3Cpro and identified potent aldehyde and α-ketoamide inhibitors. The identified inhibitors are well suited to conducting further preclinical studies to evaluate their potential as drug candidates. Furthermore, homology modeling and docking studies were conducted to illuminate the structural basis for the observed potency of the FMD 3Cpro inhibitors.

## MATERIALS AND METHODS

### Multiple sequence alignment of 3Cpro of FMDV strains

The 3Cpro sequences of 18 FMDV prototypes belonging to seven major serotypes (O, A, C, Asia 1, SAT 1–3) were obtained from the GenBank. The FMDV strains were A/IND287/96 (GenBank accession no. ACJ02492), O1/BFS 1860/UK/67 (AY593815), O1/Manisa/TUR/69 (AY593823), A21/Lumbwa/KEN/3/64 (AY593761), A22/IRQ/24/64 (AY593763), C/UK/149/34 (AY593810), C1/Santa Pau/Spain/70 (AJ133357), Asia1/PAK/1/54 (AY593795), Asia1/IND/63/72 (AY304994), Asia1/YNBS/China/58 (AY390432), SAT1/RV/11/37 (AY593839), SAT1/BEC/1/48 (AY593838), SAT1/ISR/4/62 (AY593844), SAT2/SA/106/59 (AY593848), SAT2/ZIM/7/83 (AF540910), SAT2/RHO/1/48 (AY593847), SAT3/SA/57/59 (AY593850), and SAT3/BEC/1/65 (AY593853). The FMDV 3Cpro amino acid sequences were aligned using Clustal Omega (https://www.ebi.ac.uk/Tools/msa/clustalo/) to determine amino acid homology.

### Compounds

The compounds included in this study are listed in Tables 1 to 3. Syntheses of these compounds were published elsewhere (38, 40, 45, 47). AG7088 (Rupintrivir) was purchased from MedChemExpress, LLC (Monmouth Junction, NJ).

**TABLE 1:** The IC_50_, EC_50_, and CC_50_ values of dipeptidyl (α-ketoamide or heterocycle compounds ***E6a–E8a*** [37] and tripeptidyl compounds ***E1*** [**NPI52**] and *E6* [38]) against FMDV 3Cpro in the FRET and cell-based reporter assays

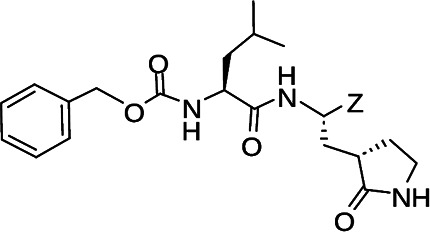				
Compound	Z	FRET assay (IC_50_, µM)^*a*^	Cell-based assay (EC_50,_ µM)^*b*^	CC_50_ (µM)
*GC373*	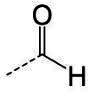	2.3 ± 0.07^*c*^	6.6 ± 0.2	>50
*E6a*	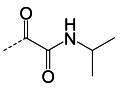	3.9 ± 0.2	NT^*d*^	>50
*E6b*	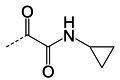	2.4 ± 0.09	8.8 ± 0.3	>50
*E6c*	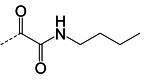	2.1 ± 0.07	7.0 ± 0.5	>50
*E6d*	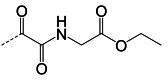	2.2 ± 0.05	9.2 ± 0.1	>50
*E6e*	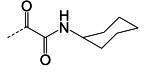	2.8 ± 0.1	NT	>50
*E6g*	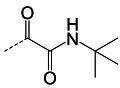	1.5 ± 0.1	5.8 ± 0.3	>50
*E8a*	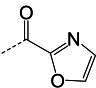	3.4 ± 0.08	8.2 ± 0.09	>50
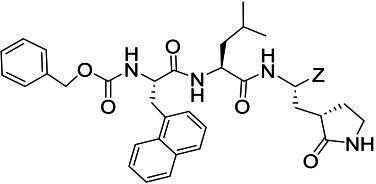				
*E 1 (NPI52*)	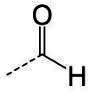	0.05 ± 0.08	0.11 ± 0.05	>50
*E6*	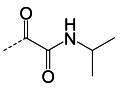	0.06 ± 0.06	0.15 ± 0.07	>50
*AG7088*	NA	4.3 ± 0.2	10.3 ± 0.2	>50

^*a*^ IC_50_, 50% inhibitory concentration.

^*b*^ EC_50_, 50% effective concentration; CC_50_, 50% cytotoxic concentration (values are M ± SD).

^*c*^ Mean (M) ± standard deviation (SD) of the means.

^*d*^ NT, not tested.

**TABLE 2:** The IC_50_, EC_50_, and CC_50_ values of cyclopropane-based compounds (***C1c/d-C17c/d*** [40]) against FMDV 3Cpro in the enzyme and cell-based report assays

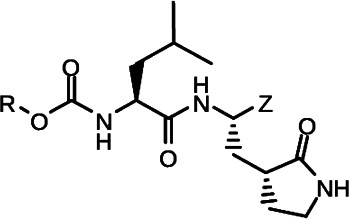					
Compound	R	Z	FRET assay (IC_50_, µM)^*a*^	Cell-based assay (EC_50_ µM)^*b*^	CC_50_ (µM)
*C1c*	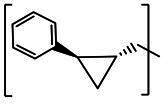	-CHO	1.00 ± 0.28^*c*^	3.15 ± 0.35	>50
*C1d*		-CH(OH)SO_3_Na	0.75 ± 0.07	3.25 ± 0.63	>50
*C2c*	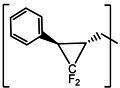	-CHO	1.35 ± 0.64	1.20 ± 0.14	>50
*C2d*		-CH(OH)SO_3_Na	1.10 ± 0.57	1.45 ± 0.21	>50
*C3c*	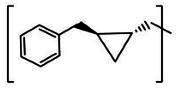	-CHO	0.69 ± 0.13	2.74 ± 2.14	>50
*C3d*		-CH(OH)SO_3_Na	0.80 ± 0.14	4.02 ± 0.30	>50
*C4c*	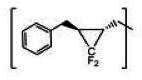	-CHO	0.82 ± 0.15	2.74 ± 2.14	>50
*C4d*		-CH(OH)SO_3_Na	1.35 ± 0.35	3.65 ± 0.63	>50
*C5c*	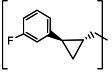	-CHO	1.05 ± 0.21	2.13 ± 0.49	>50
*C5d*		-CH(OH)SO_3_Na	0.95 ± 0.22	1.89 ± 0.09	>50
*C6c*	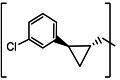	-CHO	0.80 ± 0.05	3.30 ± 0.71	>50
*C6d*		-CH(OH)SO_3_Na	0.58 ± 0.11	2.85 ± 0.49	>50
*C7c*	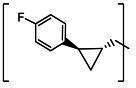	-CHO	0.59 ± 0.04	2.00 ± 0.14	>50
*C7d*		-CH(OH)SO_3_Na	0.75 ± 0.07	2.15 ± 0.35	>50
*C8c*	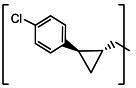	-CHO	1.15 ± 0.08	NT^*d*^	>50
*C8d*		-CH(OH)SO_3_Na	0.55 ± 0.07	NT	>50
*C9c*	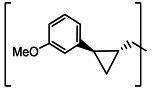	-CHO	0.75 ± 0.14	2.95 ± 0.28	>50
*C9d*		-CH(OH)SO_3_Na	0.84 ± 0.06	2.05 ± 0.64	>50
*C10c*	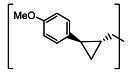	-CHO	1.25 ± 0.07	3.65 ± 1.48	>50
*C10d*		-CH(OH)SO_3_Na	1.05 ± 0.21	2.85 ± 1.55	>50
*C11c*	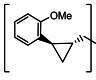	-CHO	0.95 ± 0.21	1.08 ± 0.05	>50
*C11d*		-CH(OH)SO_3_Na	0.45 ± 0.13	1.02 ± 0.17	>50
*C12c*	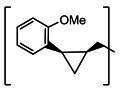	-CHO	0.50 ± 0.14	0.88 ± 0.25	>50
*C12d*		-CH(OH)SO_3_Na	0.51 ± 0.06	1.25 ± 0.07	>50
*C13c*	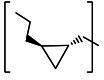	-CHO	1.55 ± 0.07	2.95 ± 0.21	>50
*C13d*		-CH(OH)SO_3_Na	0.75 ± 0.07	3.10 ± 0.42	>50
*C14c*	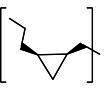	-CHO	0.63 ± 0.10	7.05 ± 0.78	>50
*C14d*		-CH(OH)SO_3_Na	1.05 ± 0.64	6.30 ± 0.85	>50
*C15c*	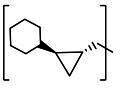	-CHO	0.38 ± 0.11	0.45 ± 0.05	>50
*C15d*		-CH(OH)SO_3_Na	0.29 ± 0.08	0.50 ± 0.11	>50
*C16c*	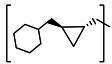	-CHO	0.36 ± 0.09	0.90 ± 0.06	>50
*C16d*		-CH(OH)SO_3_Na	0.33 ± 0.04	1.21 ± 0.48	>50
*C17c*	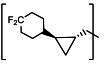	-CHO	0.65 ± 0.07	>10	>50
*C17d*		-CH(OH)SO_3_Na	0.75 ± 0.09	>10	>50

^*a*^ IC_50_, 50% inhibitory concentration.

^*b*^ EC_50_, 50% effective concentration; CC_50_, 50% cytotoxic concentration (values are M ± SD).

^*c*^ Mean (M) ± standard deviation (SD) of the means.

^*d*^ NT, not tested.

**TABLE 3:** The IC_50_, EC_50_, and CC_50_ values of compounds with a gem-dimethyl group (***D1c/d-D17c/d*** [47]) against FMDV 3Cpro in the enzyme and cell-based reporter assays

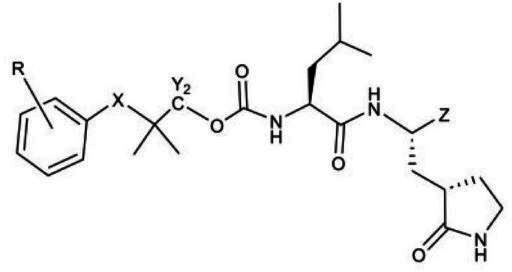							
Com	R	X	Y	Z	FRET assay (IC_50_, µM)^*a*^	Cell-based assay (EC_50_, µM)^*b*^	CC_50_ (µM)
*D1c*	H	S	H	-CHO	1.20 ± 0.71^*c*^	1.85 ± 0.35	>50
*D1d*				-CH(OH)SO_3_Na	1.01 ± 0.42	1.50 ± 0.43	>50
*D1e*				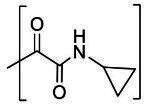	3.75 ± 0.07	5.00 ± 0.28	>50
*D1f*				-CN	>25	>10	>50
*D2c*	H	S	D	-CHO	1.52 ± 0.02	2.50 ± 0.42	>50
*D2d*				-CH(OH)SO_3_Na	1.15 ± 0.07	2.91 ± 0.43	>50
*D3c*	H	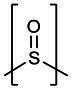	H	-CHO	1.14 ± 0.08	>10	>50
*D3d*				-CH(OH)SO_3_Na	1.05 ± 0.21	>10	>50
*D4c*	H	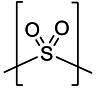	H	-CHO	0.90 ± 0.28	>10	>50
*D4d*				-CH(OH)SO_3_Na	0.65 ± 0.09	>10	>50
*D5c*	m-F	S	H	-CHO	3.15 ± 0.49	1.00 ± 0.28	>50
*D5d*				-CH(OH)SO_3_Na	0.81 ± 0.07	0.08 ± 0.14	>50
*D6c*	p-F	S	H	-CHO	0.91 ± 0.15	5.15 ± 0.64	>50
*D6d*				-CH(OH)SO_3_Na	0.98 ± 0.11	5.85 ± 0.35	>50
*D7c*	m-Cl	S	H	-CHO	1.50 ± 0.28	1.05 ± 0.21	>50
*D7d*				-CH(OH)SO_3_Na	0.60 ± 0.01	1.20 ± 0.14	>50
*D8c*	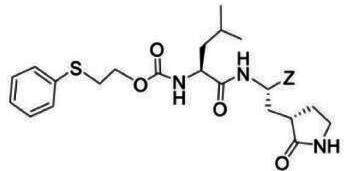			-CHO	1.35 ± 0.35	1.75 ± 0.21	>50
*D8d*				-CH(OH)SO_3_Na	1.03 ± 0.24	1.95 ± 0.22	>50
*D9c*	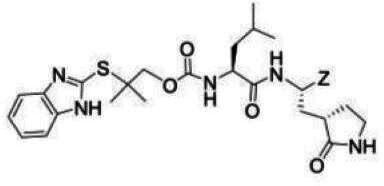			-CHO	2.15 ± 0.34	>10	>50
*D9d*				-CH(OH)SO_3_Na	1.75 ± 0.92	>10	>50
*D10c*	H	O	H	-CHO	0.74 ± 0.08	NT^*d*^	>50
*D10d*				-CH(OH)SO_3_Na	0.70 ± 0.28	NT	>50
*D11c*	p-Cl	O	H	-CHO	0.69 ± 0.06	0.64 ± 0.15	>50
*D11d*				-CH(OH)SO_3_Na	0.49 ± 0.05	0.98 ± 1.11	>50
*D12c*	p-Cl	O	D	-CHO	0.14 ± 0.02	0.35 ± 0.10	>50
*D12d*				-CH(OH)SO_3_Na	0.19 ± 0.01	0.43 ± 0.31	>50
*D13c*	m-Cl	O	H	-CHO	0.67 ± 0.08	5.34 ± 1.19	>50
*D13d*				-CH(OH)SO_3_Na	0.65 ± 0.21	7.70 ± 0.85	>50
*D14c*	m-Cl	O	D	-CHO	0.50 ± 0.14	3.38 ± 0.39	>50
*D14d*				-CH(OH)SO_3_Na	0.65 ± 0.07	4.81 ± 3.06	>50
*D15c*	p-F	O	H	-CHO	0.45 ± 0.07	5.25 ± 0.64	>50
*D15d*				-CH(OH)SO_3_Na	0.55 ± 0.07	5.90 ± 0.58	>50
*D16c*	m-F	O	H	-CHO	0.78 ± 0.03	4.90 ± 0.37	>50
*D16d*				-CH(OH)SO_3_Na	0.69 ± 0.02	5.05 ± 0.21	>50
*D17c*	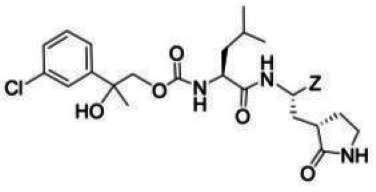			-CHO	2.00 ± 0.71	NT	>50
*D17d*				-CH(OH)SO_3_Na	0.90 ± 0.28	NT	>50

^*a*^ IC_50_, 50% inhibitory concentration.

^*b*^ EC_50_, 50% effective concentration; CC_50_, 50% cytotoxic concentration (values are mean ± SD).

^*c*^ Mean (M) ± standard deviation (SD) of the means.

^*d*^ NT, not tested.

### The FRET assay

#### Expression of recombinant FMDV 3Cpro

To express recombinant FMDV 3Cpro, the full sequence of 3Cpro of A/IND287/96 strain (GenBank accession no. ACJ02492, serotype A) encoding 213 amino acids and N-terminal 6 His tags was codon optimized for protein expression in *E. coli* and synthesized by Integrated DNA Technologies (Coralville, IA). The synthesized DNA was cloned into pET-28a(+) vector (Addgene, Cambridge, MA), and the recombinant 3Cpro was expressed in *E. coli* BL21 cells (Invitrogen, Carlsbad, CA) grown in Luria-Bertani broth by induction with 1 mM isopropyl β-D-thiogalactopyranoside. The recombinant proteins of 16.5 kDa were then purified using HIS Gravitrap Ni-NTA affinity columns (GE Healthcare, Chicago, IL) following the standard protocol (35).

#### Activity of FMDV 3Cpro and inhibition assay

To confirm the activity of 3Cpro, the expressed 3Cpro was serially diluted in assay buffer (50 mM NaCl, 6 mM dithiothreitol, 50 mM HEPES, 0.4 mM EDTA, and 60% glycerol at pH 8.0) and mixed with FRET substrate, Edans-APAKQLLN-Dabcyl (AnaSpec, Fremont, CA), and the mixture was added into a black 96-well microplate (Fisher Scientific, Waltham, MA). The plate was then measured for fluorescence at excitation and emission values 360 and 460 nm, respectively, on a fluorescence microplate reader (FLx800, Biotek, Winnooski, VT) for up to 90 min. After the activity of the expressed 3Cpro was confirmed, potency of the compounds was determined against FMDV 3Cpro, as previously described by us (35, 48). Briefly, each compound was serially diluted in DMSO or media and incubated with FMDV 3Cpro in assay buffer at room temperature (RT) for 30 min. Then, the FRET substrate was added to the mixture in a black 96-well microplate. Following incubation of the plate at RT for 30 min, fluorescence was measured on the microplate reader, and the relative fluorescence was calculated by subtracting the values for substrate-only control from the raw values. The 50% inhibitory concentration (IC_50_) was calculated using the non-linear regression analysis with four parameter variable slope in GraphPad Prism software version 9 (GraphPad Software, La Jolla, CA), as previously described (35, 48).

### Cell-based reporter assay

#### Generation of plasmids encoding the FMDV 3Cpro and circular, permutated form of firefly luciferase

The full-length 3Cpro of FMDV A/IND287/96 strain was codon optimized for protein expression in mammalian cells and synthesized by Integrated DNA Technologies. The gene was then cloned into pcDNA3 H2B-mIFP T2A vector (Addgene, Watertown, MA). The resulting plasmid was designated as pcDNA3-FMDV-3Cpro. The pGloSensor-30F-DEVDG plasmid encodes a circular, permutated form of firefly luciferase gene containing a caspase 3/7 cleavage site sequence and was obtained from Promega (Madison, WI) (49). The cleavage sequence was swapped with an FMDV 3Cpro cleavage sequence (APAKQLLN) using a QuikChange II Site-directed mutagenesis kit (Agilent, Santa Clara, CA), and the resulting plasmid was designated as pGlo-FMDV. The pGloSensor-30F plasmid contains Renilla luciferase gene as an expression control

#### Cell-based reporter assay

**Fig 1:**
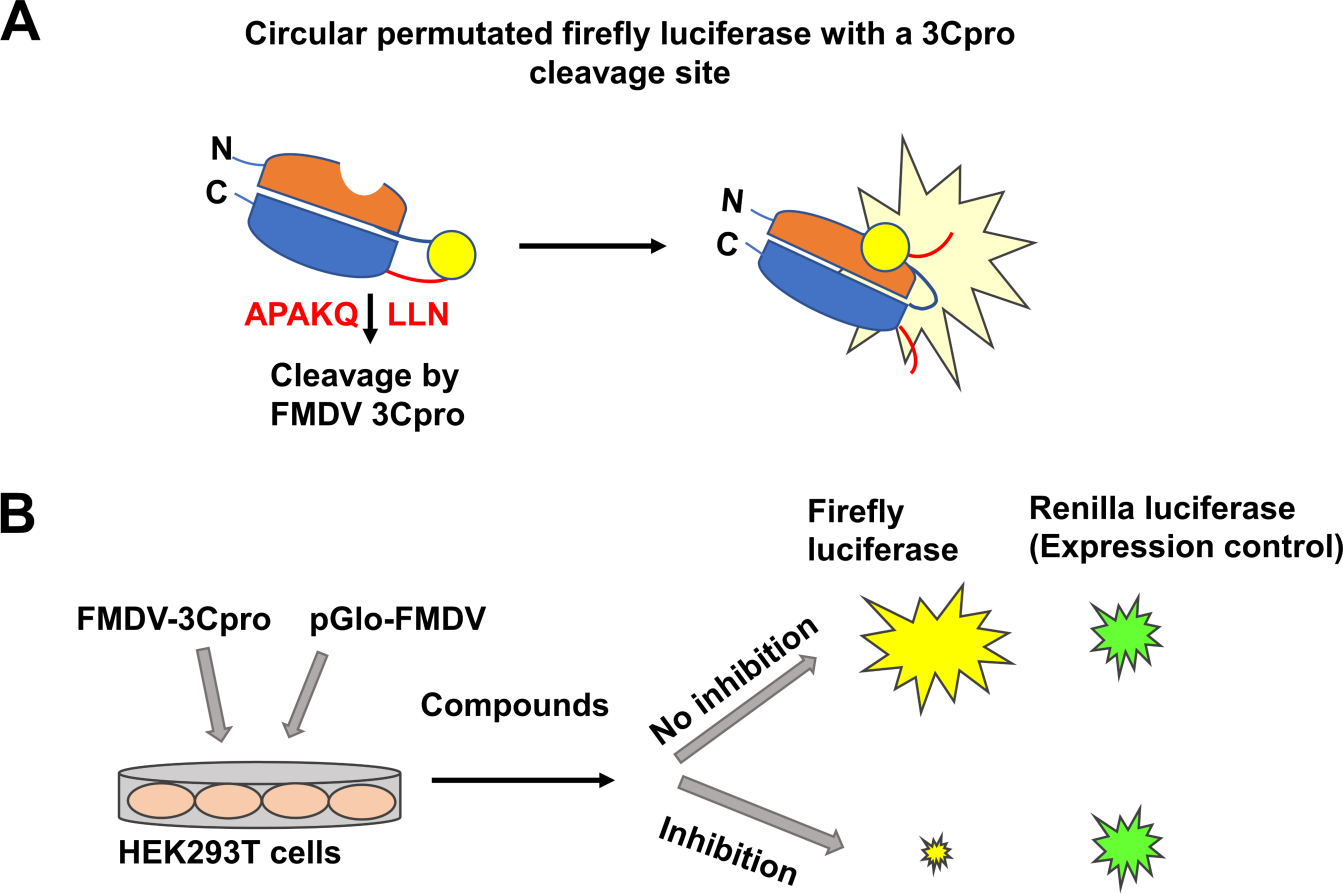
A cell-based reporter assay for screening FMDV 3Cpro inhibitors in HEK293T cells. (**A**) A plasmid encoding the permutated firefly luciferase with an FMDV 3Cpro cleavage sequence (pGlo-FMDV). The pGlo-FMDV plasmid also contains Renilla luciferase gene as an expression control. Cleavage of pGlo-FMDV by FMDV 3Cpro results in firefly luminescence. (**B**) Trypsinized HEK293T cells were electroporated with plasmids encoding FMDV 3Cpro and pGlo-FMDV and subsequently incubated with various concentrations of each compound. Following overnight incubation of the cells, luminescence was measured, and firefly luminescence was normalized against Renilla luminescence to determine the 50% effective concentration of each compound.

Trypsinized HEK293T cells were electroporated with pGlo-FMDV and pcDNA3-FMDV-3Cpro (10 ng of each plasmid) using a Neon Electroporation system (Thermo Fisher, Chicago, IL). Electroporation of pGlo-FMDV alone resulted in minimal background luminescence at 24 h after electroporation. After electroporation, cells were incubated with DMSO (0.1%) or each compound at 10, 2, 0.5, 0.1, and 0.02 µM for 20 h. Following lysis of the cells, firefly and Renilla luciferases were measured using a dual luciferase reporter assay (Promega, Madison, WI) on a luminometer (GloMax 20/20 Luminometer, Promega), following the manufacturer’s direction. Firefly luciferase was normalized against Renilla luciferase, and the 50% effective concentration (EC_50_) of each compound was calculated by GraphPad Prism software. Figure 1B illustrates the cell-based reporter assay.

### Cytotoxicity assay

To determine the cytotoxicity of the compounds, 70%–80% confluent HEK293T cells in a 96-well plate were incubated with serial dilutions of each compound (up to 50 µM) for 24 h. Cell cytotoxicity was determined by a CytoTox 96 nonradioactive assay kit (Promega) by measuring cytosolic enzyme lactate dehydrogenase, following the manufacturer’s instructions. The 50% cytotoxic concentration (CC_50_) value of each compound was calculated using GraphPad Prism software. The non-specific cytotoxic effects of these compounds were also reported previously by us (35, 38, 40, 45, 47).

### Three-dimensional modeling of FMDV 3Cpro bound with inhibitor *D12c*

The binding mode of ***D12c*** in the active site was modeled using the coordinates of a previously determined 3Cpro crystal structure of FMDV A serotype (Protein Data Bank accession no. 2BHG) by superimposing ***D12c*** in the active site of 3Cpro. The ***D12c***-bound 3Cpro model was prepared for docking by adding the covalent bond between ***D12c*** and the sulfur atom (Sg) of catalytic residue Cys163 and specifying His46 as the epsilon 2 nitrogen (HIE) tautomer with protonation of the nitrogen (Ne) atom. The protein preparation wizard in Schrodinger was used to optimize hydrogen bonding and minimize the structure, using Schrodinger’s OSPL4 energy function (50), and ***D12c*** was prepared for docking using LigPrep (50). These models were subsequently used for covalent docking using CovDock, also from Schrödinger (50, 51), selecting the “Nucleophilic Addition to a Double Bond” reaction, performing MM-GBSA scoring with flexibility within 6 Å, and outputting five poses per ligand reaction site.

## RESULTS

### Multiple amino acid alignment of 3Cpro of various FMDV serotypes

**Fig 2:**
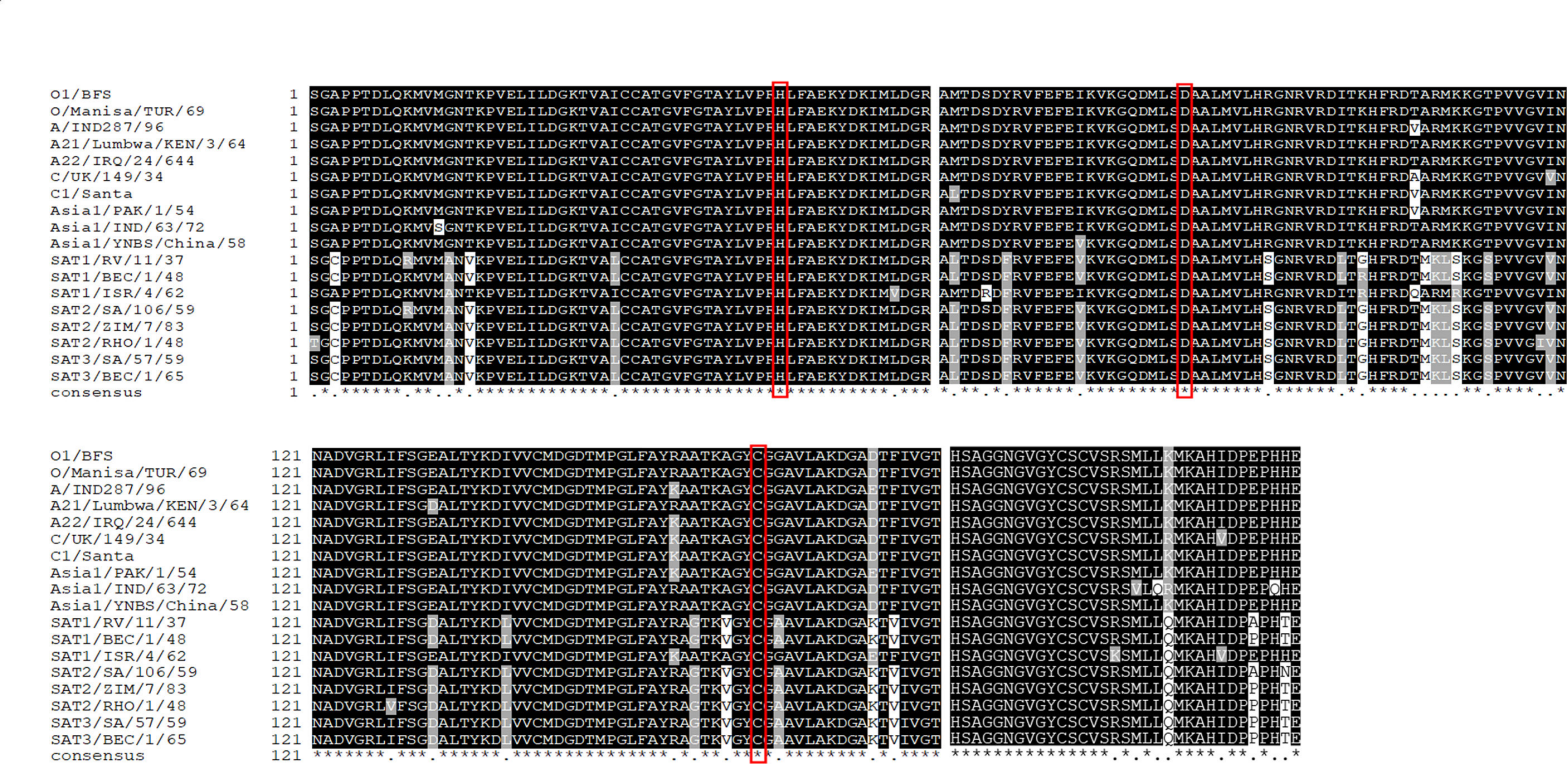
Multiple alignment of 3Cpro amino acid sequences of 18 FMDV prototype strains belonging to seven serotypes (O, A, C, Asia1, and SAT1–3). The conserved catalytic residues in the active site of 3Cpro are shown in red boxes.

The homology of 3Cpro amino acid sequences of the reference strains of FMDV serotypes was 84.51%–100% with conserved active site residues (His46, Asp84, and Cys163) (Fig. 2). FMDV serotypes A, O, and C and Asia1 share high 3Cpro homology at greater than 96% and a relatively lower homology with serotypes SAT1-3 at 85.45%–86.85%. The 3Cpro sequences are highly conserved with greater than 98% homology among SAT1–3 serotypes.

### Activity of compounds against FMDV 3Cpro in the FRET assay

**Fig 3:**
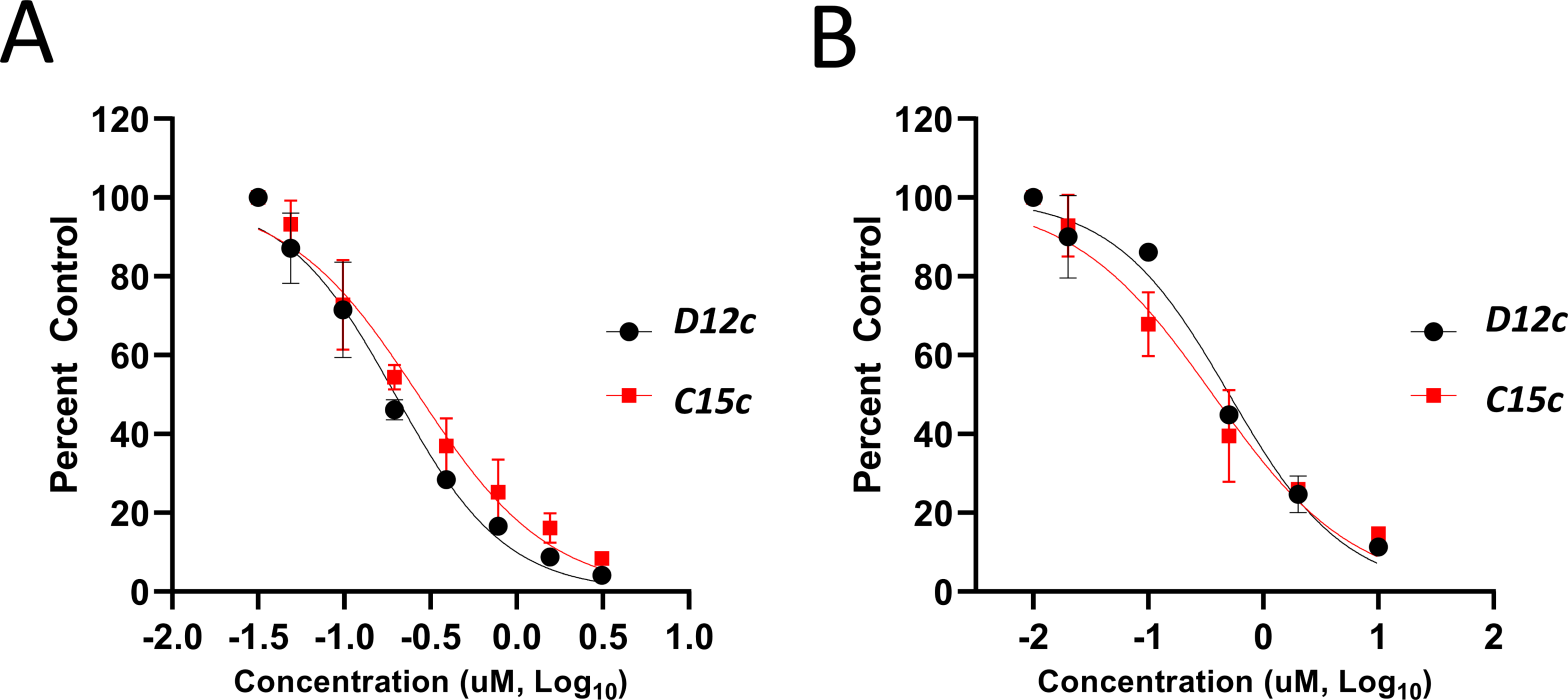
Dose-dependent inhibition curves of ***C15c*** and ***D12c*** from the FRET assay (**A**) and the cell-based reporter assay (**B**). Values are expressed as mean ± standard deviation of the means from at least three independent experiments.

Aldehyde compound GC373 and its α-ketoamide or heterocycle dipeptidyl derivatives, which were previously reported to have activity against norovirus 3CLpro (45), inhibited FMDV 3Cpro with IC_50_ values >1 µM (Table 1). All dipeptidyl compounds with an α-ketoamide or α-heterocycle warhead had IC_50_ values comparable to GC373, ranging from 1.5 to 3.9 µM. Tripeptidyl compounds ***E1*** **(NPI52**) and ***E6*** showed strong inhibitory activity with 0.05 and 0.06 µM IC_50_ values, respectively (Table 1). AG7088, a tripeptidyl inhibitor of human rhinovirus 3Cpro, was less potent than tripeptidyl compounds ***E1*** (***NPI52***) and ***E6*** and most tested dipeptidyl compounds. The anti-FMDV 3Cpro activity of cyclopropane-based compounds (40) and gem-dimethyl compounds, which were recently reported to be potent coronavirus (SARS-CoV-2 and MERS-CoV) 3CLpro inhibitors (47), was also determined (Tables 2 and 3). All tested cyclopropane-based inhibitors in Table 2 showed good inhibitory activities (IC_50_ values of 0.29–1.55 µM) against FMDV 3Cpro. As expected, the aldehyde and bisulfite adduct pairs showed similar activities. Among cyclopropane-based inhibitors, ***C15c/C15d*** pair was most potent with IC_50_ values of 0.38 and 0.29 µM, respectively (Table 2). The potency of compounds with a gem-dimethyl group ranged from 0.14 to 3.75 µM, and among them, the ***D12c/D12d*** pair was most potent with IC_50_ values of 0.14 and 0.19 µM, respectively (Table 3). The dose-dependent inhibition curves of ***C15c*** and ***D12c*** in the FRET assay are shown in Fig. 3A.

### Activity of compounds against FMDV 3Cpro in the cell-based reporter assay

The EC_50_ values of selected compounds were determined in this assay (Tables 1 to 3). All dipeptidyl compounds with an α-ketoamide or α-heterocycle warhead had EC_50_ values comparable to GC373 and ranged from 5.8 to 9.2 µM (Table 1). When a tripeptidyl compound with aldehyde (***E1***) or a ketoamide warhead (***E6***) (Table 1) was tested, they displayed good inhibitory activity with EC_50_ values of 0.11 and 0.15 µM, respectively (Table 1). The EC_50_ value of AG7088 was determined to be 10.3 µM. The EC_50_ values of the compounds in Tables 2 and 3 ranged from 0.35 to 7.7 µM. Among the tested dipeptidyl compounds in this assay system, ***D12c*** exhibited the highest potency with an EC_50_ value of 0.35 µM, which is consistent with the result from the FRET assay. The dose-dependent inhibition curves for ***C15c*** and ***D12c*** from the cell-based report assay are shown in Fig. 3B. All the compounds that were included in the study showed minimal cytotoxicity at up to 50 µM in HEK-293T cells (Tables 1–3).

### Modelling of the binding mode of *D12c* to FMDV 3Cpro

**Fig 4:**
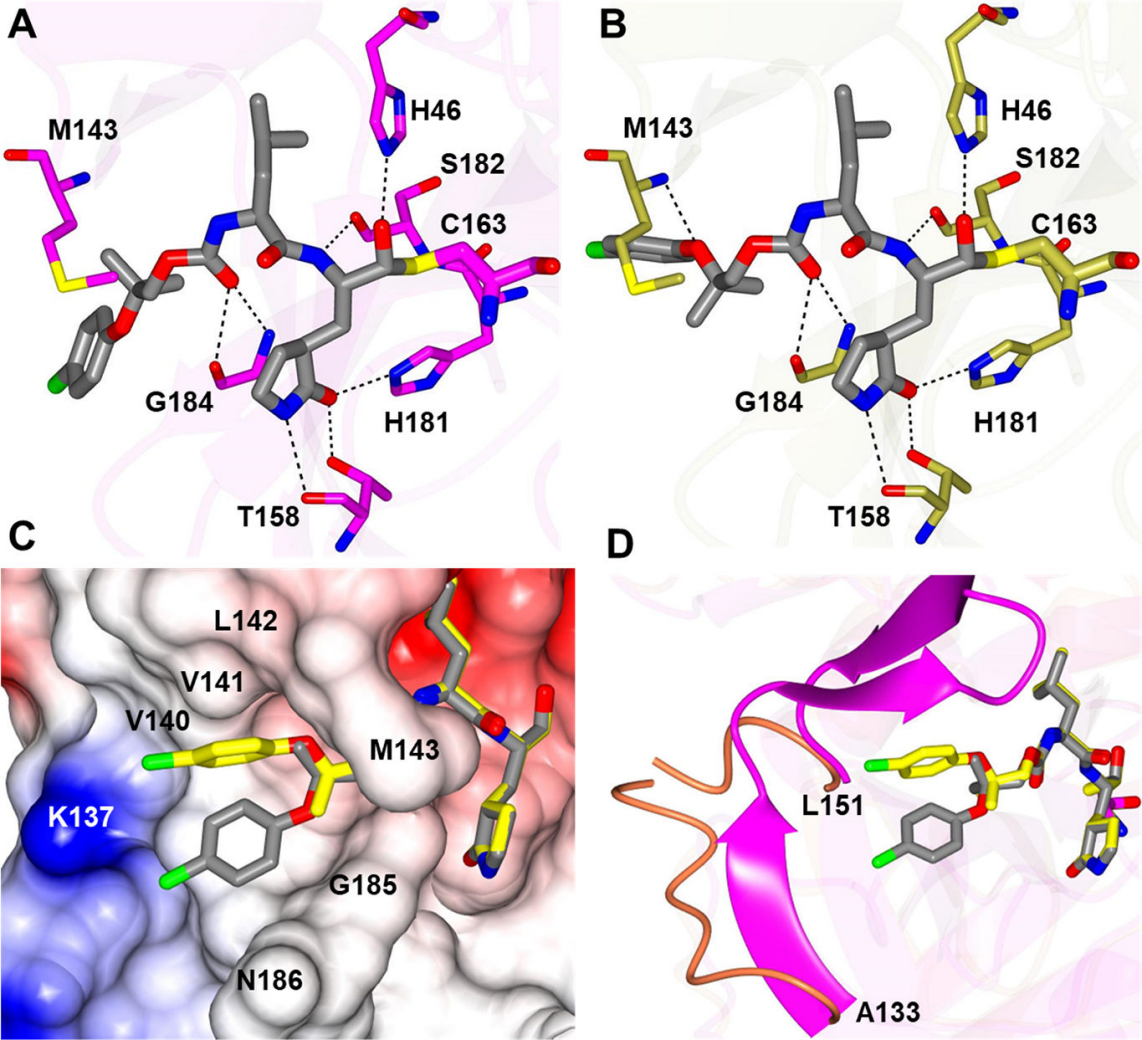
Modeling of ***D12c*** in the active site of FMDV 3Cpro. (**A and B**) Hydrogen bond interactions (dashed lines) for poses 1 and 2, respectively. (**C**) Electrostatic surface representation showing the putative orientation of *p*-chlorophenyl ring in the S4 subsite. (**D**) Superposition of ***D12c*** and the crystal structure of FMDV 3Cpro (Protein Data Bank 2BHG, magenta) highlights the differences in the loop spanning A133-L151. The poses 1 and 2 of ***D12c*** are colored gray and yellow, respectively.

The top 2 poses of the ***D12c*** adopt similar conformations in the FMDV 3Cpro active site where hydrogen bond interactions are formed between H46, T158, S182, H181, S182, and G184 (Fig. 4A and B). The main difference between the two poses is in the orientation of the *p*-chlorophenyl ring. The *p*-chlorophenyl ring of one pose is positioned away from M143 (pose 1), whereas the other forms a hydrogen bond with the backbone N-atom of M143 (pose 2). For both poses, the *p*-chlorophenyl ring is positioned in a hydrophobic cleft as shown in Fig. 4C. Comparison with the crystal structure of FMDV 3Cpro (Protein Data Bank 2BHG) revealed some interesting differences. Superposition (47) of the ***D12c***-bound structure with the apo structure of FMDV 3Cpro is similar overall with a root mean square deviation between Ca atoms at 1.10 Å (182 residues). The main difference is observed in the loop spanning A133-L155, which is disordered in 2BHG but adopts a β-sheet fold in the ***D12c***-bound model, where the loop moves toward the inhibitor to form a hydrogen bond with the backbone nitrogen of M143 in pose 2 and also engages in hydrophobic interactions (Fig. 4D).

## DISCUSSION

The FMD genome encodes a single large polyprotein, which is proteolytically cleaved at 10 out of 13 cleavage sites by 3Cpro into functional viral proteins. This virally encoded, indispensable protease is highly conserved structurally and functionally among FMDV serotypes, which makes it an attractive target for the development of antivirals to formulate an additional layer of mitigation strategy, which can complement and strengthen the existing control measures for FMD outbreaks. Although limited number of studies are available on virus- or host-targeting antiviral agents of FMDV (9, 16, 17, 20, 22, 23, 26, 27, 52–55), some studies showed that pegylated recombinant interferon and T-1105 (polymerase inhibitor) reduced or prevented viremia in FMDV-challenged pigs (22, 52) and GS-9620, an agonist of toll-like receptor 7, enhanced survival rate in FMDV-vaccinated and un-vaccinated mice (16). Compounds that are shown to have *in vitro* activity against FMDV with EC_50_ values of low micromolar to millimolar range include polymerase inhibitors 5D9 (20, 23), 24a (2-amino-4-arylthiazole derivatives) (24) and ribavirin (24), and a rhinovirus entry inhibitor pleconaril (17). These results, especially the *in vivo* data, support the potential use of antivirals in immediate emergency response as well as prophylactic treatment, but several issues, such as serotype/genotype-wide efficacy, pharmacokinetics, and potential combined use of different classes of antiviral agents, are needed to be addressed.

The overall designing of 3Cpro or 3CLpro inhibitors from our previous studies has primarily utilized a known peptidyl recognition element attached to various warheads, such as an aldehyde, α-keto heterocycle, or α-ketoamide, in the case of transition-state analog inhibitors, which interact with the active site Cys to yield a tetrahedral adduct (35, 53, 56–58). The warhead can also be a Michael acceptor, such as an α,β-unsaturated ester or vinyl sulfone which, in contrast to aldehydes and α-ketoamides, reacts with the active site Cys to form a covalently bound enzyme inhibitor adduct (59–65). The most extensively studied inhibitors of this class (Michael acceptors) are AG7088 and its variants, which have been shown to be active against various picornaviruses, including human rhinoviruses (59, 63, 66), enterovirus-D68 (61), and FMDV (25). The *in vitro* potency of AG7088 against FMDV was above 10–20 µM EC_50_ (25) and is comparable to the EC_50_ value obtained from the cell-based reporter assay reported herein.

We previously reported that compounds with an α-ketoamide warhead are slightly less active against calicivirus or coronavirus 3CLpros compared to their aldehyde counterparts (35, 45, 47, 67). We have also shown that compounds with an α-ketoamide warhead is highly effective against picornavirus 3Cpro and comparable to aldehyde counterparts (46). When the di- and tripeptidyl compounds with various ketoamide residues were evaluated to assess the effects of α-ketoamide warhead on FMDV 3Cpro, both compound series showed similar potency with α-ketoamide or aldehyde warheads (Table 1), which was similar to what we observed with picornavirus 3Cpros. Among the compound series evaluated in this study, the most potent compounds against FMDV 3Cpro were ***E1*** (***NPI52***) and ***E6*** with EC_50_ values 0.11 and 0.15 µM, respectively (Table 1). For these tripeptidyl compounds including ***E1*** and ***E6***, we previously reported that they are highly effective against calicivirus and coronavirus 3CLpros (34, 38), but they might not have favorable *in vivo* pharmacokinetics (34).

The cyclopropane-based inhibitors (Table 2) were recently reported by us (40) as highly potent inhibitors against 3CLpros of SARS-CoV-2, SARS-CoV, and MERS-CoV. In this study, it was shown that the cyclopropane-based inhibitors also have activity against FMDV 3Cpro with varying potency. Structure-activity relationship analysis of the series revealed that replacement of the methylene group in the cyclopropane ring with a gem-difluoro group did not increase potency (***C1c/d*** vs ***C2c/d***) (Table 2). Among the halogen substituted compounds, potency was also invariant to the nature and position of halogen substitution in the phenyl ring (compounds ***C5c/d*** through ***C8c/d***), and these compounds were also similar to the corresponding unsubstituted compound (***C1c/d***) in potency (Table 2). Furthermore, the isomeric methoxy-substituted phenyl compounds (***C9c/d**, **C10c/d***, and ***C11c/d***) were fairly effective against FMDV 3Cpro in the FRET assay (Table 2). However, replacement of the benzene ring with the cyclohexane ring increased potency by approximately twofold (***C1c/d*** vs ***C15c/d***). Among this series of inhibitors, ***C15c/C15d*** were most potent against FMDV 3Cpro with IC_50_ and EC_50_ values of 0.29–0.38 µM and 0.45–0.50 µM, respectively (Table 2). Compounds ***C15c/C15d*** also have activity against SARS-CoV-2 replicon in cell culture, but the most potent pairs against SARS-CoV-2 were ***C5c/d*** and ***C11c/d*** (EC_50_ 0.01–0.03 µM) (40), which suggests some differences in structural requirements for potency between these two viruses.

The second series of inhibitors (47) exploited the directional effects associated with the presence of a *gem*-dimethyl group that allowed the inhibitors to optimally interact with the S4 subsite of 3Cpro. The EC_50_ values of aldehyde ***D1c*** and its corresponding bisulfite adduct ***D1d*** against SARS-CoV-2 were 0.012 and 0.010 µM, respectively (47). Previously, we reported that replacement of sulfur by oxygen (Table 3, X position) had varying effects in potency against 3CLpros of SARS-CoV-2 (decreased potency) and MERS-CoV (increased potency) (47). In the case of FMDV 3Cpro, replacement of sulfur by oxygen yielded more potent compounds (Table 3, ***D1c/d*** vs ***D10c/d***; ***D5c/d*** vs ***D16c/d***; ***D6c/d*** vs ***D15c/*d**; and ***D7c/d*** vs ***D13c/d***). Interestingly, replacement of the aldehyde warhead with an α-ketoamide made no difference in potency against FMDV 3Cpro (***D1c*** vs ***D1e***), while the same replacement reduced potency against SARS-CoV-2 3CLpro (47). In addition, replacement with a nitrile warhead (***D1f***) diminished activity against FMDV 3Cpro at up to 25 µM (Table 3). When non-deuterated and deuterated pairs were compared for potency, some pairs showed unchanged activity (Table 3, ***D1c/d*** vs ***D2c/d*** and ***D11c/d*** vs ***D12c/d***), while deuteration of ***D11c/d*** to ***D12c/d*** moderately increased potency by approximately twofold (Table 3), making the ***D12c/d*** pair the most potent one against FMDV 3Cpro.

Among the compounds in Tables 2 and 3, the most potent compounds against FMDV 3Cpro were ***D12c*** and ***D12d*** with IC_50_ values 0.14 and 0.19 µM, respectively (Table 3), which is comparable to those with ***E1*** and ***E6*** (Table 1). Generally, IC_50_ values were comparable to EC_50_ values for each compound; however, some compounds showed variations between IC_50_ and EC_50_ values, especially the gem dimethyl series (Table 3), with better potency in the enzyme assay compared to the cell-based assay. The variation might be due to the cell permeability and stability of each compound, which highlights the importance of a cell-based assay for evaluating antiviral activity.

Recently, two papers (26, 27) by Theerawatanasirikul et al. and a paper by Lee et al. (9) showed natural products (including quercetin, luteolin, and isoginkgetin) and compounds (NSC116640 and NSC332670) from a chemical library inhibited the replication of FMDV in cell culture via inhibiting 3Cpro, and the molecular docking studies suggested that these compounds fit in the active site and interact with key residues of FMDV 3Cpro. In this study, the binding mode of ***D12c*** in the active site of FMDV 3Cpro was modeled by superimposing the compound on a previously determined crystal structure of 3Cpro. The non-deuterated counterpart of ***D12c*** [compound ***11d*** in Fig. 8A–C in reference (47)] was previously co-crystalized with SARS-CoV-2 3CLpro to identify the structural determinants associated with the binding of the inhibitor to the active site of SARS-CoV-2 3CLpro. The modeling shows that the five-ring glutamine surrogate tightly sites at S1 position with three hydrogen bonds with H181 and T158 of FMDV 3Cpro. In addition, hydrogen bonds are predicted between ***D12c*** and H46 and S182 and G184 of FMDV 3Cpro, which may explain the high potency of the compound. For *p*-chlorophenyl ring of ***D12c***, the modeled structure of FMDV 3Cpro and the co-crystal structure of SARS-CoV-2 bound to the inhibitor (47) showed that the residue has a similar orientation in the S4 subsite of both enzymes.

Screening many antiviral compounds against FMDV replication can be challenging as it requires biosafety level (BSL)-3E or 3Ag facility, and Plum Island Animal Disease Center is the only research facility in the US that can conduct research involving live virus. Thus, we established the FRET and cell-based reporter assays which can be used in BSL-2 laboratories, which can facilitate the drug discovery process for FMDV. In summary, we conducted structure-activity relationship studies on multiple series of protease inhibitors for FMDV 3Cpro using the FRET and the cell-based reporter assays and identified potent compounds with double-digit or triple-digit nanomolar EC_50_ values. The homology modeling and docking study revealed an insight into the binding mechanism between a 3Cpro inhibitor and FMDV 3Cpro. The identified inhibitors of FMDV 3Cpro warrant conducting further multiparameter optimization studies to identify one or more lead candidates for drug development for prophylactic and treatment options in immediate emergency response.

## Data Availability

The detailed chemical structures are shown in Tables 1 to 3, and the detailed information on the synthesis of listed chemicals was reported in references 38, 40, 45, 47.
